# Turning their backs on the ‘ladder of success’? Unexpected responses to the MacArthur Scale of Subjective Social Status

**DOI:** 10.12688/wellcomeopenres.18655.2

**Published:** 2023-03-15

**Authors:** Rachael H. Moss, Brian Kelly, Philippa K. Bird, Hannah Z. Nutting, Kate E. Pickett

**Affiliations:** 1 Bradford Institute for Health Research, Bradford Teaching Hospitals Foundation Trust, Bradford, West Yorkshire, BD9 6RJ, UK; 2 Leeds Teaching Hospitals NHS Trust, Leeds, West Yorkshire, LS9 7FT, UK; 3 Department of Health Sciences, University of York, York, North Yorkshire, YO10 5DD, UK

**Keywords:** Subjective status, MacArthur Scale of Subjective Social Status

## Abstract

Subjective social status measures a person’s perception of their social class relative to other people and has theoretically and empirically been positively associated with health and wellbeing. A widely used measure of this construct is the MacArthur Scale of Subjective Social Status, which asks people to report their social status by placing themselves on a ladder which represents the social hierarchy of their society or community; the scale has been used with many different populations across many countries. In this research note, we describe two cases where we encountered unexpected reactions to the MacArthur Scale that we believe highlight (a) the salience of relative social status for people’s wellbeing in contemporary society and (b) the concomitant sensitivities raised by measuring this subjective experience. We discuss the implications of these observations for future research.

## Introduction

**Figure 1.**
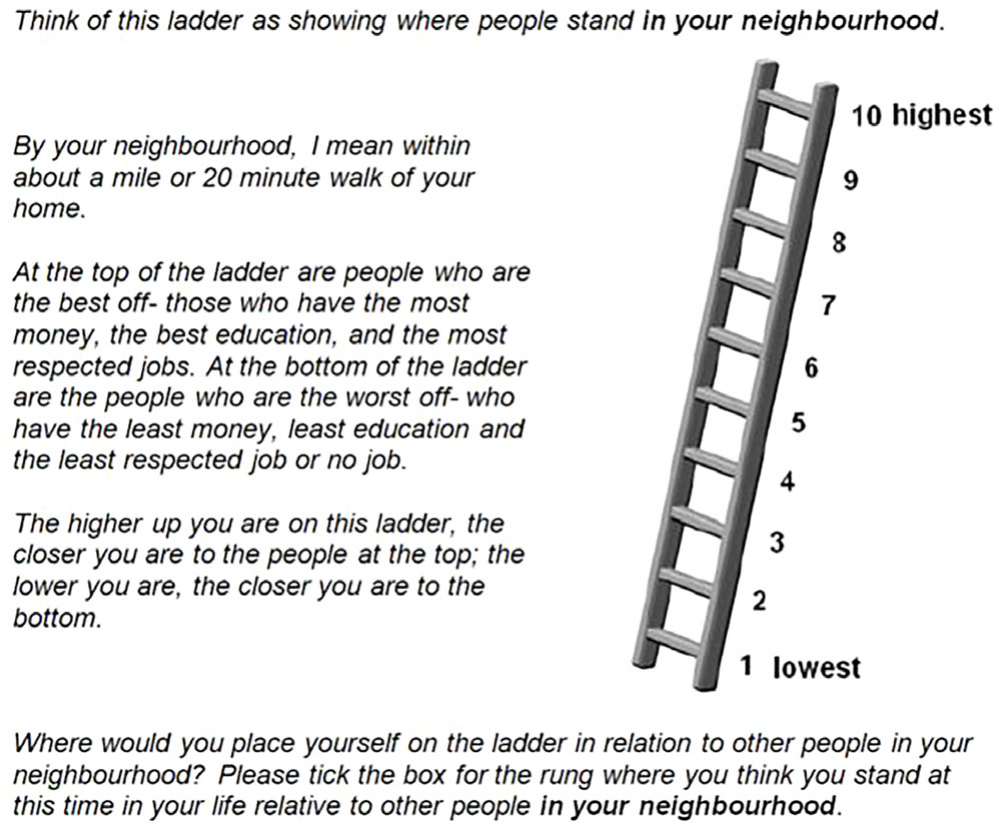
A copy of the MacArthur Scale used within the baseline BiBBS (Born in Bradford’s Better Start) cohort survey. This question asks about subjective social status in relation to other people in your neighbourhood.

Subjective social status captures a person’s perception of their social class relative to other people and has theoretically and empirically been positively associated with health and wellbeing. Ladders are widely used as a metaphor for social class ranking in both lay discourse and scholarly work ^1^ and the MacArthur Scale of Subjective Social Status aims to capture people’s overall sense of where they stand within the social hierarchy of their society using an image of a ladder with 10 rungs, numbered from 1 at the bottom to 10 at the top ( Figure 1). People are asked to place themselves on the ladder according to their social status in relation to others, within their country and/or within their neighbourhood or community. The scale was developed by Adler *et al.*, ^2^ within the Research Network on Socioeconomic Status and Health, supported by the MacArthur Foundation from 1996 to 2009. The English language version of the scale gives descriptions of the social status of people at the top of the ladder (these people are the ‘best off’ - they ‘have the most money, the most education and the best jobs’) and at the bottom (these people are the ‘worst off’ - they ‘have the least money, least education and the worst jobs or no job’). Respondents are asked to place an ‘X’ on the rung that best represents where they think they stand on the ladder. When used with young people, in addition to being asked to rank themselves within their society, they are also asked to rank themselves on a ladder that represents the social hierarchy within their school.

The MacArthur Scale has been widely used in epidemiological and public health research and is robustly associated with health outcomes. Subjective social status is associated with health similarly to objective measures, such as education, occupational prestige and income, and it has therefore been viewed as a useful proxy for objective social status in situations where study populations might not know or wish to report their incomes. In addition, it has also been shown to be associated with health after adjusting for objective measures, supporting the theory that subjective social status is a concept of value in itself for the understanding of population health and wellbeing ^3, 4^. There is substantial literature attesting to the validity and reliability of the MacArthur Scale and it has been translated into several languages. To our knowledge, there are no other measures of subjective social status in general use in population health research.

The aetiological roles of objective vs. subjective measures of social status, and underlying theories about causal pathways within the social determinants of health framework, continue to be researched (see for example 5) and are matters of debate. However, in this research note we report an issue encountered in our contemporary research: how people respond to being asked to report their subjective social status when presented with the MacArthur Scale of Subjective Social Status in two separate studies and discuss what their responses mean for health research.

## Responses to proposed use of the MacArthur Scale of Subjective Social Status in a contemporary cohort study of children and young adults

### Ethics

Ethical approval for Age of Wonder, including the co-production process, has been granted by NHS Leeds Bradford Research Ethics committee (Approval number ref: 21/YH/0261, date 22.12.21). Regulatory approval has been granted by the Health Research Authority.

Written informed consent for publication of the participants’ details was obtained from the participants themselves. For reference, please refer to the following reference: Dickerson J, Bird PK, McEachan RR, Pickett KE, Waiblinger D, Uphoff E, Mason D, Bryant M, Bywater T, Bowyer-Crane C, Sahota P, Small N, Howell M, Thornton G, Astin M, Lawlor DA, Wright J. Born in Bradford’s Better Start: an experimental birth cohort study to evaluate the impact of early life interventions. BMC Public Health. 2016 Aug 4;15(1):711. doi: 10.1186/s12889-016-3318-0. PMID: 27488369; PMCID: PMC4996273. The Age of Wonder qualitative protocol can be found here for reference also: Dogra, S. A., Lightfoot, K., Kerr, R., Hall, J., Joseph, O., Siddig, N., … & Wright, J. (2022). Born in Bradford Age of Wonder cohort: A protocol for qualitative longitudinal research. Wellcome Open Research, 7(270), 270. doi: 10.12688/wellcomeopenres.18096.1.”

Born in Bradford (BiB) is a population-based birth cohort study of 13,818 children born between 2007 and 2011 and their families ^6^. BiB participants are currently transitioning to secondary school, and the Wellcome Trust have funded a new wave of research (BiB Age of Wonder) that will follow the children and young people through to age 21 years. Age of Wonder has a strong focus on participant engagement; throughout 2021 we conducted focus groups and workshops with young people to co-produce the survey questionnaires that will be used in the next few years ^7^.

The aim of Age of Wonder is to understand the causes and consequences of health and wellbeing at key life transitions into adolescence and adulthood, collecting information on family, lifestyle, environmental, cognitive, social and health exposures and outcomes. The MacArthur Scale was presented to young people for comment on its potential inclusion in the survey to measure subjective social status.

As part of this co-production process, we received consistently negative comments and concerns in regards to the MacArthur Scale items. Young people expressed concern about being “uncomfortable or embarrassed” about having to answer these items, with another stating that such items were “divisive”. Some stated that they get compared enough in everyday life, “without putting it on a ladder”. Young people felt that being asked to respond to the ladder question would encourage them to “talk about it and compare themselves”, something that one young person stated is “the opposite of what school encourages us to do - not to compare yourself with others”. Several individuals suggested adding a message about the sensitive nature of the topics being asked at the beginning of the survey, to acknowledge that some may find these questions “triggering”. These comments suggested that it was likely that the MacArthur scale items would be met with unease and discomfort if used amongst a larger group of similarly aged young people. As a result of this co-production, we decided to omit the MacArthur Scale from our Age of Wonder study.

## Responses to the MacArthur Scale of Subjective Social Status in a contemporary birth cohort study

Born in Bradford’s Better Start (BiBBS) is a population-based birth cohort study with ongoing recruitment from 2016 of pregnant women within three inner-city wards of Bradford, UK ^8^. Bradford is the 5th largest city in England, with a population of more than 530,000. The city has an ethnically diverse population, including a large Pakistani community and growing communities of East European and Roma people. It has high levels of poverty and deprivation, and the three wards included in this study (the Better Start Bradford area) are among the most deprived in Bradford district and among the 10% most deprived in England ^9^. BiBBS aims to understand the lives, relationships, wellbeing, and social and economic circumstances of pregnant women and their children.

The current release of BiBBS baseline data is a data-freeze at 30 ^th^ November 2021 ^10^, with 1,861 women in total who completed a long version of the baseline survey, generally administered at 26–28 weeks gestation, that included the MacArthur Scale of Subjective Social Status (measuring the social status of individuals in relation to both their Bradford community and England as a whole). The sample includes 1,763 women who had one pregnancy, 97 women with two pregnancies and one woman with three pregnancies since January 2016. Ethical approval for recruitment and collection of baseline and routine outcome data and biological samples for the cohort has been approved by Bradford Leeds NHS Research Ethics Committee (15/YH/0455). Research governance approval has been provided from Bradford Teaching Hospitals NHS Foundation Trust.

Whilst conducting an analysis of participants’ responses to the ladder questions (paper forthcoming), we noticed a higher non-response rate than we typically receive within our studies in Bradford. When presented with the ladder asking them to rate their social status relative to England on a larger scale, 16% responded that they did not wish to answer, and 1.3% had a missing response; for the ladder which asked participants to compare themselves to other people in their neighbourhood, 13.3% responded that they did not wish to answer and 1.3% had a missing response.

To explore the possibility that survey fatigue was leading to a high non-response rate, we looked at the responses to questions that came just before, and just after, the MacArthur scale items. For both the preceding question about self-reported financial status and the following question about family relationships, rates of non-response were much lower than for the ladder questions ( Table 1).

**Table 1.** Response rates to questions within the Born in Bradford’s Better Start baseline survey (n=1,861 women).

Survey question	Do not wish to answer	Missing	Total non- response
**Immediately preceding MacArthur Scale**			
How well would you say you (and your partner) are managing financially these days?	2.3%	0.5%	**2.8%**
Compared to a year ago, how would you say you (and your partner) are doing financially now?	3.6%	0.5%	**4.1%**
**MacArthur Scale of Subjective Social Status**			
Where would you place yourself on the ladder in relation to other people in your neighbourhood?	13.3%	1.3%	**14.6%**
Where would you place yourself on the ladder in relation to other people in England?	16.0%	1.3%	**17.3%**
**Immediately following MacArthur Scale**			
I feel closely attached to my family	2.5%	1.4%	**3.9%**
My family take notice of my opinions	3.1%	1.4%	**4.5%**
Sometimes I feel excluded in my own family	4.7%	1.4%	**6.1%**

Participants appear to be selectively choosing not to complete one or both of the ladder items, whereas they almost always choose to answer questions about self-reported financial status and family relationships, items that may be deemed as being sensitive in nature. As the MacArthur Scale has been widely used and validated throughout the world it seems unlikely that participants found the questions difficult to answer.

## Discussion

In summary, we found that there was a high level of non-response specific to the MacArthur scale questions amongst an ethnically diverse population of pregnant women in Bradford, and that young people involved in a co-production process in Bradford provided an overwhelmingly negative response towards the use of the MacArthur Scale of Subjective Social Status. Taken together, these findings suggest that contemporary health research studies may face a challenge in (a) trusting the validity of responses to the measure if rates of missing data are high, and (b) measuring subjective social status at all.

Our participants’ reactions and responses to the MacArthur Scale may be telling us about the high prevalence of the social pain of subjective social status in contemporary society. Literature illustrates that social pain activates the same neural substrates as physical pain ^11^ – it really does hurt - and that experiencing ‘social evaluative threat’ (being exposed to the negative judgement of others) is associated with heightened hormonal stress responses ^12^. Further research is needed to explore reactions to, and responses to, the MacArthur Scale and to understand people’s lived experiences of relative social status and their sensitivities to being asked to compare themselves to others in this way. Some research suggests that subjective social status may vary across population groups; Shaked *et al.*, ^13^ report modifications of the effect of subjective social status on health by sex and ethnicity. It may well be that the reactions and responses we have encountered in Bradford would not be found, or would be even more pronounced, in different societies and communities.

Research is also needed to co-produce acceptable and feasible measures that can capture how individuals see themselves in relation to others. In the contemporary context of high prevalence of social media use in many age groups, this feels like an important construct to measure in studies of the psychosocial determinants of health and wellbeing, and one that should be captured in surveys and not only in in-depth qualitative studies. The very reasons why people dislike the MacArthur Scale is what makes measuring subjective and relative social status so important. Seeing ourselves through the eyes of others shapes our wellbeing ^14^, now perhaps more than any time in history, and if researchers are to be able to study the consequences of this, they need acceptable and valid measures of subjective social status.

## Data Availability

Researchers are encouraged to make use of the BiBBS data, which are available through a system of managed open access. Before you contact us, please make sure you have read our Guidance for Collaborators. Our BiB Executive reviews proposals on a monthly basis and we will endeavour to respond to your request as soon as possible. You can find out about the different datasets in our Data Dictionary. If you are unsure if we have the data that you need please contact a member of the BiB team ( borninbradford@bthft.nhs.uk). Once you have formulated your request, please complete the ‘Expression of Interest’ form available here and send to borninbradford@bthft.nhs.uk. If your request is approved, we will ask you to sign a Data Sharing Contract and a Data Sharing Agreement, and if your request involves biological samples we will ask you to complete a material transfer agreement.
