# Supplementary material for: Turning their backs on the ‘ladder of success’? Unexpected responses to the MacArthur Scale of Subjective Social Status
Source: Wellcome Open Res. 2023 Mar 15;8:11. Originally published 2023 Jan 6. [Version 2] doi: 10.12688/wellcomeopenres.18655.2 (PMC9971696; doi:10.12688/wellcomeopenres.18655.2)
Supplement: Peer review history

# Peer review: Reviewer response for version 1

**DOI:** 10.21956/wellcomeopenres.20685.r54079

**Published:** 2023-02-09

**Authors:** Anna Barford, Kate Brockie

**Affiliations:** 1 Murray Edwards College, University of Cambridge, Cambridge, UK; 2 Department of Geography, University of Cambridge, Cambridge, UK

The wrong tool for the job? Ladders as a measure of subjective social status.

 The main contribution of the research note is to identify the limitations of the widely used MacArthur scale of subjective social status. The authors identify that self-perceived social status has profound impacts on individuals’ physical and mental health, so finding appropriate ways to measure this subjective trait is consequently of great importance. This scale requests respondents to position themselves on a scale/ladder from rank/rung one to ten - the higher the number the higher their perceived social status.

 With evidence from two different groups in the Born in Bradford study, the authors show how participants had distinct reservations when responding to this measure in research. Criticism of the MacArthur scale’s use in the study was demonstrated by young people advising against its use in a forthcoming study, and as mothers-to-be voting with their feet by not answering this question as part of a longer survey. The potential inability of the MacArthur scale to ethically and accurately capture subjective social status, as showcased in these two observations, is the motivation for writing the research note. 

 Another contribution was the focus on engaging young people in the co-production of a survey. The MacArthur scale was shared with young people as an option for inclusion in the survey; their critical feedback resulted in this scale being excluded from the survey design. Given our interest in engaging young people in research, we were pleased to learn of this co-produced survey for the ‘Age of Wonder’ study. This is especially interesting in a context when much youth-involved research to date has been qualitative (e.g. Proefke and Barford, forthcoming ^1^). 

 Overall the paper is well written, insightful, useful, and easy to understand. We have a few suggestions for improvements. In the abstract it is worth specifying the type and level of responses to the MacArthur scale which were observed, instead of referring somewhat vaguely to ‘unexpected reactions’. This clarification would help communicate your key messages more strongly within the abstract. More generally, the paper could be improved by stronger signposting of the key message(s) throughout, rather than leaving this as something to be discovered in the discussion section. One place to do this would be adding a sentence or two at the end of the introduction. It would also help if the authors could clearly state early on that there are two data sources referred to in this note – the co-production of a survey with young people and an earlier survey with mothers, and clarify the connection between these groups. Also, perhaps also rewrite this clause in simpler language: “The current release of BiBBS baseline data is a data-freeze at 30th November 2021”.

 To extend the thinking and reflection on the authors’ observations, we would like to share a few thoughts. Firstly, it is worth questioning the suitability of a ladder for depicting socio-economic hierarchies. Arguably, the ladder is misleading. A ladder suggests something to be climbed (up or down), fitting into political discourses focused on social mobility and opportunity (and avoiding discussions of actual outcomes; see Barford, 2016 ^2^). This comes with the implied degree of responsibility for one’s own position, irrespective of the wider structural limitations/advantages that people face. The ladder also seems to depoliticise inequalities because it doesn’t convey the proportion of the population who would be found at each rung, and implies that movement between rungs is equally easy (e.g. no recognition of glass ceilings).

 The authors could usefully consider visual alternatives to the ladder which would demonstrate how being on the lower rung would mean being with the majority, and which could provoke less shame about positioning oneself low down (in the same way that the occupy and 99% movements were based on solidarity and shared disadvantage). A pyramid could be one way to do this (e.g. Barford and Ahmad, 2021, figure 2 ^3^). Nancy Krieger (Krieger, 2008 ^4^) has a useful paper on iconographies of inequality. Ha-Joon Chang (Chang, 2002 ^5^) elaborates on the ladder metaphor to critique how wealthy countries have blocked the economic development of poorer countries, in his book ‘Kicking Away the Ladder.’

 Another consideration is about what the MacArthur scale is really getting at. Respondents are asked to reflect upon their social status based on their job, income and education. Arguably these are relatively objective measures - one’s income, job, and level of education are factual and measurable. Yet these are used to access self-perceptions of social status, something which has many more than three dimensions. In Keith Payne’s book ‘The broken ladder’ (p.13) (Payne, 2017 ^6^) he suggests that only 20% of the self-evaluation of social status is based on income, education and job status. The authors could make reference to research into the discrepancies between subjective social status and objective markers (e.g. Adler *et al.*, 2000 ^7^).

 We would be delighted to see the authors consider the reasons for these negative reactions from the two groups with whom they trialled the MacArthur ladder. The paper considers social evaluative threat in the discussion; could this be heightened and more painful precisely because the scale requires respondents to internalise external judgements and then report on themselves? The request to rank oneself demands the dropping one’s guard, breaking into any protective defensiveness about one's own social positioning. It would also be interesting to know whether there was a pattern to rejecting the MacArthur scale question. It is possible that some groups are more uneasy about this question than others; could the authors analyse patterns of non-responses in the second study according to other characteristics? Looking ahead, would different imagery result in different levels of engagement, and how might this vary between groups? And of course, we are curious to know what will be missed by excluding this measure from the ‘Age of Wonder’ study? What information could this have provided you with, and why was the measure of interest for the Born in Bradford research in particular? 

 Finally, as times change, sensitivity to the question of social status may have increased. Could this measure have become outdated - or was it always problematic? As we embark upon 2023, the ‘Age of Wonder’ cohort have almost certainly been exposed to considerable posturing and displays of wealth (via social media) compared to previous generations. As such, the reference group for the Born in Bradford cohorts when identifying their position in wider society may include a wider spectrum of income and wealth. Having a higher status reference group may heighten the contrast in status and thus diminish respondents’ perceived social status to the extent that they would refuse to answer a question on this topic - at a time when research into inequalities and the damage they cause is highly relevant.

Is the work clearly and accurately presented and does it cite the current literature?

Yes

If applicable, is the statistical analysis and its interpretation appropriate?

Yes

Are all the source data underlying the results available to ensure full reproducibility?

Yes

Is the study design appropriate and is the work technically sound?

Yes

Are the conclusions drawn adequately supported by the results?

Yes

Are sufficient details of methods and analysis provided to allow replication by others?

Yes

Reviewer Expertise:

The reviewers have a shared interest in socio-economic inequality. Kate has expertise in social surveys and statistical analysis related to socio-economic categorisations. Anna’s PhD focused on visualisations and discourses of inequality and how people respond to these in diverse settings. Since her PhD Anna has continued to research issues of inequality, disadvantage, and interventions to address these. Both Kate and Anna share a research interest in young people, including how to involve young people in academic research.

We confirm that we have read this submission and believe that we have an appropriate level of expertise to confirm that it is of an acceptable scientific standard.

## References

[1] : Creating spaces for co-research. *Journal of the British Academy* .(forthcoming); Reference source

[2] : Discourses supporting socio-economic inequality in Kenya, Mexico and the United Kingdom. *Portuguese Journal of Social Science* .2016;15(3) :331-347 Reference source

[3] : A Call for a Socially Restorative Circular Economy: Waste Pickers in the Recycled Plastics Supply Chain. *Circ Econ Sustain* .2021;1(2) : 10.1007/s43615-021-00056-7 761-782 10.1007/s43615-021-00056-7 34888563PMC8192276

[4] : Ladders, pyramids and champagne: the iconography of health inequities. *J Epidemiol Community Health* .2008;62(12) : 10.1136/jech.2008.079061 1098-104 10.1136/jech.2008.079061 19008371

[5] : Kicking Away the Ladder: Development Strategy in Historical Perspective. *Anthem Press* .2002; Reference source

[6] : The Broken Ladder: How Inequality Affects the Way We Think, Live, and Die. *Viking* .2017;

[7] : Relationship of subjective and objective social status with psychological and physiological functioning: Preliminary data in healthy, White women. *Health Psychology* .2000;19(6) : 10.1037//0278-6133.19.6.586 586-592 10.1037//0278-6133.19.6.586 11129362

[8] : Emotional responses to world inequality. *Emotion, Space and Society* .2017;22: 10.1016/j.emospa.2016.10.006 25-35 10.1016/j.emospa.2016.10.006

# Peer review: Reviewer response for version 1

**DOI:** 10.21956/wellcomeopenres.20685.r54082

**Published:** 2023-02-02

**Authors:** Laura Howe

**Affiliations:** 1 Population Health Sciences, Bristol Medical School, University of Bristol, Bristol, UK

This is a really interesting report, which will be useful to other research teams who are thinking about capturing data on subjective social status. It is really useful to see the patterns of missing data in the MacArthur ladder variable, and how these compare with the preceding and following questions. It was also interesting to hear about the experiences in the focus groups with young people. I did wonder if it might be possible to present a bit more information about/from the focus groups - e.g. if there are any relevant quotes, or if there are any data on how many focus groups were conducted, with how many young people in each, and how many of the groups agreed with the concerns about the MacArthur ladder. I'd also be interested in how the MacArthur ladder was presented to each group, and whether the concerns about the ladder were raised spontaneously in the groups, or whether questions about potential concerns were posed to the groups. I recognise that not all of these data may be available, and would suggest that these additions would be welcome but are not essential if they are not possible.

Is the work clearly and accurately presented and does it cite the current literature?

Yes

If applicable, is the statistical analysis and its interpretation appropriate?

Not applicable

Are all the source data underlying the results available to ensure full reproducibility?

No source data required

Is the study design appropriate and is the work technically sound?

Yes

Are the conclusions drawn adequately supported by the results?

Yes

Are sufficient details of methods and analysis provided to allow replication by others?

Yes

Reviewer Expertise:

Social epidemiology

I confirm that I have read this submission and believe that I have an appropriate level of expertise to confirm that it is of an acceptable scientific standard.
